# Mesonephric carcinoma of the cervix associated with ovarian serous carcinoma: a case report

**DOI:** 10.1093/omcr/omae117

**Published:** 2024-10-15

**Authors:** Ayoub Kharkhach, Asmae Bali, Said Afqir, Tariq Bouhout, Badr Serji

**Affiliations:** Faculty of Medicine and Pharmacy, Mohammed First University, BP 724 Hay Al Quods, Oujda 60000, Morocco; Department of Surgical Oncology, Oncology Hospital, Mohammed VI University Hospital, BP 4806 Oujda Universite 60049, Morocco; Faculty of Medicine and Pharmacy, Mohammed First University, BP 724 Hay Al Quods, Oujda 60000, Morocco; Department of Medical Oncology, Oncology Hospital, Mohammed VI University Hospital, BP 4806 Oujda Universite 60049, Morocco; Faculty of Medicine and Pharmacy, Mohammed First University, BP 724 Hay Al Quods, Oujda 60000, Morocco; Department of Medical Oncology, Oncology Hospital, Mohammed VI University Hospital, BP 4806 Oujda Universite 60049, Morocco; Faculty of Medicine and Pharmacy, Mohammed First University, BP 724 Hay Al Quods, Oujda 60000, Morocco; Department of Surgical Oncology, Oncology Hospital, Mohammed VI University Hospital, BP 4806 Oujda Universite 60049, Morocco; Faculty of Medicine and Pharmacy, Mohammed First University, BP 724 Hay Al Quods, Oujda 60000, Morocco; Department of Surgical Oncology, Oncology Hospital, Mohammed VI University Hospital, BP 4806 Oujda Universite 60049, Morocco

**Keywords:** mesonephric carcinoma, ovarian serous carcinoma, debulking surgery, pathology

## Abstract

Malignant mesonephric tumor of the uterine cervix is an extremely uncommon subtype of cervical adenocarcinoma with rare, documented cases in the literature. In this report, we present a case of 58 yo, with abdominal pain and ascites that was found to have a synchronous presence of a mesonephric adenocarcinoma of the cervix and advanced serous ovarian carcinoma on the surgical specimen. The histological study identified a tumor showing a mix of tubular and ductal growth patterns. Immunohistochemical analyses were positive for cytokeratin, vimentin, calretinin and CD10. However, the tumor cells were negative for estrogen receptor and progesterone receptor. The patient received neoadjuvant chemotherapy with a combination of carboplatin and gemcitabine followed by optimal debulking surgery and was alive after 18 months of follow up. The management of this rare case remains unclear due to the absence of management guidelines.

## Introduction

Malignant mesonephric carcinomas are uncommon tumors of the female genital tract, predominantly observed in the uterine cervix [1, 2]. Their classical origin is thought to be from the embryological remnant of the Wolffian ducts, which may exist along the lateral walls of the vagina, cervix, and uterine corpus, as well as in the upper female genital tract, including the rete ovarii in the ovarian hilum or in the broad ligament [2]. Since the clinical diagnosis is similar to most uterine tumors, the histopathological features are challenging and may be misdiagnosed as other varieties of adenocarcinoma [3, 4]. Therefore, immunohistochemical and genetic investigations are necessary to define these tumors [4]. Few cases of mesonephric carcinoma have been described in literature. Here we present a new case of an interesting and rare combination of two distinct components: advanced ovarian serous carcinoma and an incidental finding of a mesonephric adenocarcinoma of the cervix on the pathological specimen. Given their scarcity in medical literature, this unique case aims at increasing awareness among clinicians and pathologists and advocate for multidisciplinary collaboration in managing such complex cases for improved patient outcomes.

## Case report

**Figure 1:**
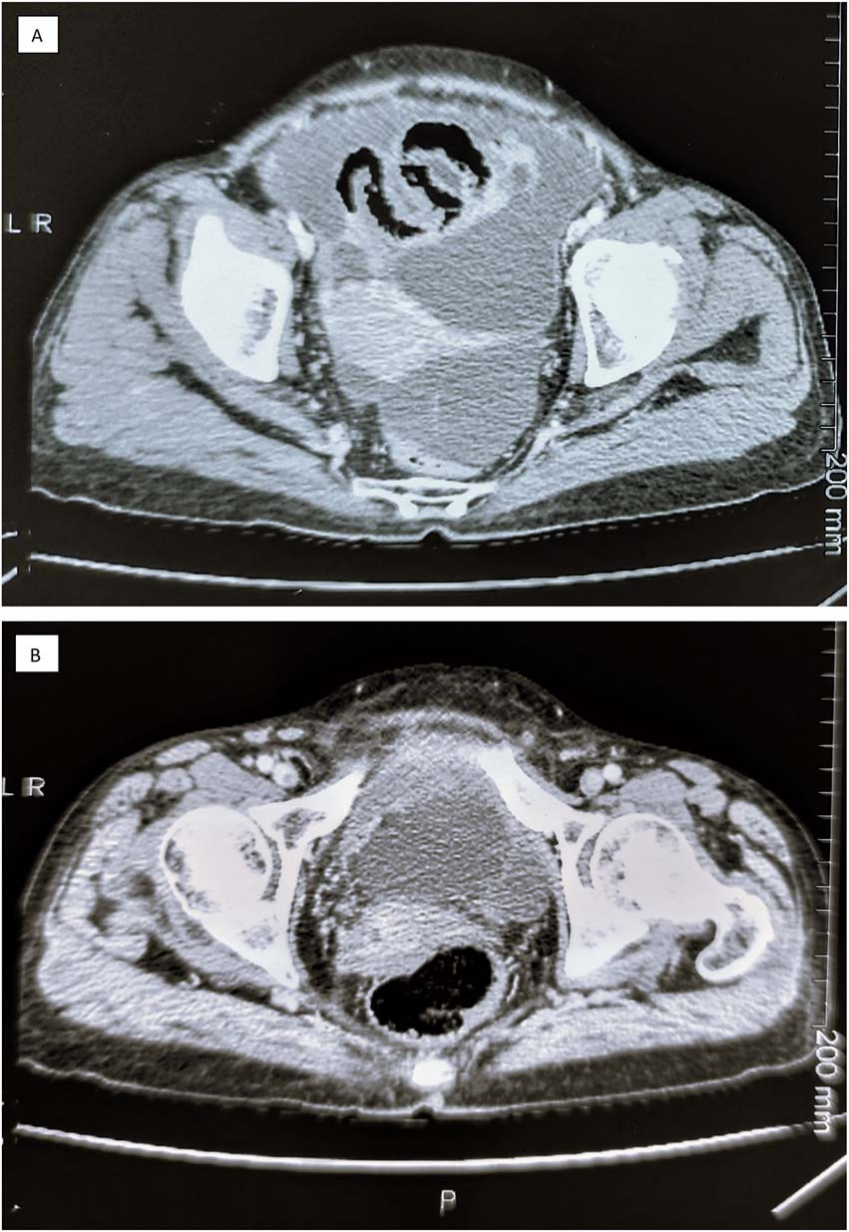
The Computed tomography of the abdomen and pelvis showing a massive ascites (**A**) with bilateral latero-uterine heterogeneous masses and peritoneal carcinomatosis (**B**).

**Figure 2:**
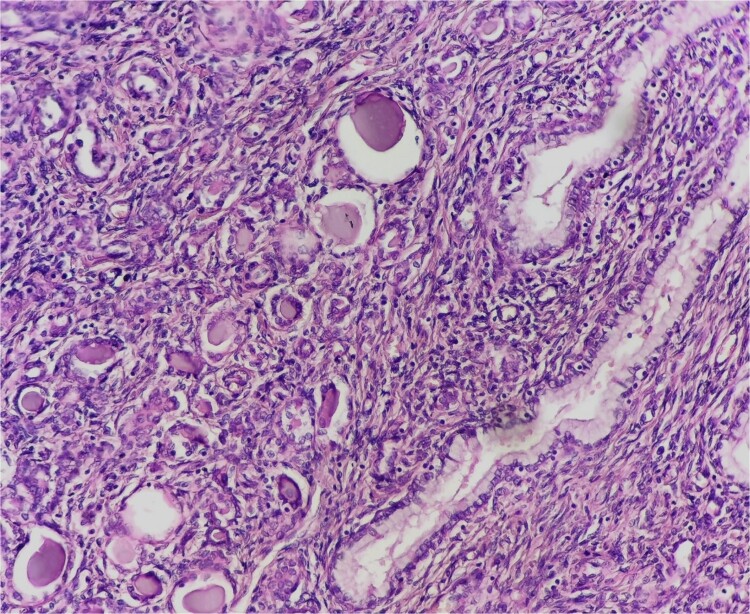
Mesonephric Adenocarcinoma of the Cervix. Epithelial neoplasm with a tubular, ductal, and papillary growth pattern producing intraluminal eosinophilic material in densely hyalinized stroma.

A 58 year old woman, G2 P2, who presented in surgical oncology department, with abdominal pain, discomfort, vomiting and weight loss of two month, no digestive or gynecological signs were noted. Physical examination revealed an abdominal distension with clinical ascites, and a shifting dullness in the abdomen, bimanual pelvi-rectal examination was normal, and no palpable lymphadenopathy was noticed. The laboratory test found that Cancer antigen 125 (CA 125) was elevated at 96 U/ml. The computed tomography of the abdomen and pelvis displayed massive ascites with bilateral latero-uterine heterogeneous masses of 82 × 50 mm and peritoneal carcinomatosis (Fig. 1). The patient underwent an exploratory surgery with peritoneal biopsies that revealed a metastatic undifferentiated ovarian carcinoma with immunostaining of epithelial membrane antigen (EMA) and cytokeratin 7 (CK7), and negative immuno-reactivity of WTT, CK 20, PAX8 and hormonal receptors (estrogen and progesterone receptors). The patient received five cycles of conventional treatment in combination with carboplatin and gemcitabine every 3 weeks (q3w) for a period of 3 months. The patient’s CA-125 levels declined to 8.46 U/ml, and the abdominal distension and pain were alleviated after the third cycle of chemotherapy. The patient underwent a debulking surgery with hysterectomy, a bilateral salpingo-oophorectomy, omentectomy, appendicectomy and removed all evidence of gross disease. The pathological examination shows a tumor that was confined to the myometrium of uterine corpus and cervix with carcinomatous growth patterns characterized by large sheets of small round tubules containing densely eosinophilic secretions. It exhibited a combination of tubular and ductal structures, along with cribriform formations. Immunohistochemical analysis revealed positive staining for cytokeratin, vimentin, calretinin, CD10 with luminal staining, while testing negative for estrogen receptor and progesterone receptor. These findings were indicative of a mesonephric origin tumor (Fig. 2). According to the Tumor-Nodes-Metastasis (TNM) classification of malignant tumors, it was classified pT1b Nx M1 with the presence of lympho-vascular invasion but no perineural invasion. In addition to that, a serous adenocarcinoma component was seen in both fallopian tubes and ovaries. Following these results, the patient was referred for chemotherapy where she received a single cycle of adjuvant chemotherapy with the same prior regimen, but it was stopped because of thrombocytopenia. The patient was free of disease after a follow up of 18 months.

## Discussion

Mesonephric adenocarcinoma is a rare subtype of uterine cervix tumors, which is not related to human papilloma virus [4, 5]. It represents a minority of tumors, comprising less than 1% of all cases in this location, and they are thought to derive from vestigial remnants of the mesonephric ducts [3, 6]. In adults, mesonephric remnants can be found in up to 22% of them while the prevalence can reach 40% in children [5, 7]. The median age of presentation is 53 years, with no apparent peak as it could be observed in women from third to seventh decade. The majority of mesonephric carcinoma cases are found in the uterine cervix, but it has been reported that it can also arise in the ovaries, vagina, and uterine corpus [8, 9]. Hence, hyperplasia and florid mesonephric remnants are frequently observed in the genesis of mesonephric carcinoma [10]. However, mesonephric hyperplasia, in contrast to its malignant counterpart, is usually an accidental finding and does not require therapy [3]. Interestingly, coexistent gynecologic lesions such as leiomyoma, adenomyosis, biphasic variants with sarcomatoid features, or biphenotypic tumors of serous carcinoma and mesonephric carcinoma were reported in previous reports [1–3]. As far as we know, this is the first case of a concomitant existence of these two different types of malignancies reported in the literature. Histopathologically, the mesonephric adenocarcinoma is usually infiltrative, it is predominantly characterized by a tubular and ductal growth pattern, as well as, papillary, retiform, and glomeruloid architecture. The tubular pattern shows closely packed small round glands lined by low cuboidal cells, containing dense eosinophilic secretions. In contrast, the ductal pattern displays large glandular spaces with occasional intraluminal intussusceptions or papillae, lined by columnar cells with hyperchromatic nuclei [3, 5]. The immunohistochemical profile of cervical mesonephric carcinomas usually shows a diffuse positive immunostaining for epithelial markers such as CK7 [1, 3]. However, the negative immunoreactivity for estrogen and progesterone receptors and carcinoembryonic antigen are considered crucial features that could distinguish the mesonephric carcinoma from differential diagnosis, particularly, endometrioid adenocarcinoma [1, 4, 5]. In our case, in addition to the mesonephric carcinoma located in the uterine cervix, an ovarian low grade serous carcinoma component was found on the specimen. This surprising discovery was a challenge to explain and to make a therapeutic decision afterwards. Nevertheless, mutations in genes such as KRAS, as well as low tumor mutation burden can also be seen [2, 5]. These genetic features of mesonephric carcinoma were found to be shared with serous carcinoma as demonstrated by David et al. and Kiyong et al. [1, 2]. Accordingly, it can be admitted that mesonephric carcinoma and serous carcinoma share the same origine and may arise from pluripotent stem cells with the ability to differentiate into both serous and mesonephric cell lineages as suggested by David et al. [2]. For most reported cases, the prognosis of mesonephric carcinoma seems to be worse than other type of cervical carcinomas even when low stage [1, 5]. The Progression-free survival is up to 10 months [3]. Nevertheless, local recurrence and distant metastases could reach 32% in patients with stage I [10]. Moreover, advanced International Federation of Gynecology and Obstetrics (FIGO) stage, elevated mitotic activity, and lymphovascular invasion were identified as independent factors predicting the development of metastasis in the series of Kiyong et al [1]. Notably the majority of patients succumbed within a year following recurrence [3]. In the lack of definite recommendations about treatment of mesonephric carcinoma, it is advised to manage this rare situation in accordance with the latest recommendations for cervical adenocarcinoma with comparable stage and pathological characteristics [4]. Yet, our patient received neoadjuvant chemotherapy for advanced ovarian cancer since the diagnostic of mesonephric carcinoma has been established later on the specimen of the second look surgery. However, this report may also have some limitations as our patient was not adequately followed up and cycles of chemotherapy were not optimally administered due to COVID-19 outbreak conditions.

## Conclusion

To the best of our knowledge, this is the first case of concomitant presence of ovarian serous carcinoma with mesonephric carcinoma of the cervix. This rare entity has a distinctive pathological and immunophenotype. Recent reports in literature, based on the study of genetic features by NGS, suggest a probable unique origine of mesonephric carcinoma and serous carcinoma. Effective chemotherapy followed by surgery was performed in our case with favorable outcomes. Further studies of large cohorts are necessary to establish accurate recommendation in similar situations.

## CRediT statement

All authors contributed toward data analysis, drafting and revising the paper, gave final approval of the version to be published and agreed to be accountable for all aspects of the work.
